# Histone deacetylase inhibitors valproate and trichostatin A are toxic to neuroblastoma cells and modulate cytochrome P450 1A1, 1B1 and 3A4 expression in these cells

**DOI:** 10.2478/v10102-009-0019-x

**Published:** 2009-09-28

**Authors:** Jana Hřebačková, Jitka Poljaková, Tomáš Eckschlager, Jan Hraběta, Pavel Procházka, Svatopluk Smutný, Marie Stiborová

**Affiliations:** 1 Department of Pediatric Hematology and Oncology, 2^nd^ Medical School, Charles University and University Hospital Motol, Prague 5, Czech Republic; 2 Department of Biochemistry, Faculty of Science, Charles University, Albertov 2030, 128 40 Prague 2, Czech Republic; 3 1^st^ Department of Surgery, 2^nd^ Medical School, Charles University and University Hospital Motol, V Úvalu 84, 150 06 Prague 5, Czech Republic

**Keywords:** histone deacetylase inhibitors, valproate, trichostatin A, neuroblastoma, cytotoxicity

## Abstract

Histone deacetylase inhibitors such as valproic acid (VPA) and trichostatin A (TSA) were shown to exert antitumor activity. Here, the toxicity of both drugs to human neuroblastoma cell lines was investigated using MTT test, and IC50 values for both compounds were determined. Another target of this work was to evaluate the effects of both drugs on expression of cytochrome P450 (CYP) 1A1, 1B1 and 3A4 enzymes, which are known to be expressed in neuroblastoma cells. A malignant subset of neuroblastoma cells, so-called N-type cells (UKF-NB-3 cells) and the more benign S-type neuroblastoma cells (UKF-NB-4 and SK-N-AS cell lines) were studied from both two points of view. VPA and TSA inhibited the growth of neuroblastoma cells in a dose-dependent manner. The IC_50_ values ranging from 1.0 to 2.8 mM and from 69.8 to 129.4 nM were found for VPA and TSA, respectively. Of the neuroblastoma tested here, the N-type UKF-NB-3 cell line was the most sensitive to both drugs. The different effects of VPA and TSA were found on expression of CYP1A1, 1B1 and 3A4 enzymes in individual neuroblastoma cells tested in the study. Protein expression of all these CYP enzymes in the S-type SK-N-AS cell line was not influenced by either of studied drugs. On the contrary, in another S-type cell line, UKF-NB-4, VPA and TSA induced expression of CYP1A1, depressed levels of CYP1B1 and had no effect on expression levels of CYP3A4 enzyme. In the N-type UKF-NB-3 cell line, the expression of CYP1A1 was strongly induced, while that of CYP1B1 depressed by VPA and TSA. VPA also induced the expression of CYP3A4 in this neuroblastoma cell line.

## Introduction

Neuroblastoma is the major cause of death from neoplasia in infancy (Maris and Mathay, 1999). These solid extracranial tumors are biologically heterogeneous, with cell populations differing in their genetic programs, maturation stage and malignant potential (Brodeur, 2003). Low-risk neuroblastoma is one of the rare human malignancies that are known to demonstrate spontaneous regression in infants from an undifferentiated state to a completely benign cellular formation (ganglioneuroma), whereas high-risk neuroblastoma grows relentlessly and may be rapidly fatal. Prognosis of high-risk form of cancer is poor, because drug resistance arises in the majority of those patients, initially responding to chemotherapy (Brodeur, 2003).

Neuroblastoma consists of two principal neoplastic cells (Voigt *et al*., 2000; Hopkins-Donaldson *et al*., 2004): i) neuroblastic or N-type: undifferentiated, round and small scant cytoplasm cells; and ii) stromal or S-type: large hyaline, flattened and adherent differentiated cells. As neuroblastoma cells seem to have the capacity to differentiate spontaneously *in vivo* and *in vitro* (Morgenstern *et al*., 2004), their heterogeneity could affect treatment outcome, in particular the response to apoptosis induced by chemotherapy.

To achieve the most suitable concept of treatment, drugs are usually used in various combinations. Agents commonly used in neuroblastoma treatment are platinum compounds (cisplatin, carboplatin), alkylating agents (cyclophosphamide, ifosfamide, melphalan), topoisomerase II inhibitors (etoposide), anthracycline antibiotics (doxorubicin) and vinca alkaloids (vincristine). Some novel regimen include also topoisomerase I inhibitors (topotecan and irinotecan) that are effective against recurrent disease (Brodeur, 2003).

Because the epigenetic structure of DNA and its lesions play a role in the origin of human neuroblastomas, pharmaceutical manipulation of the epigenome may offer other treatment options also for neuroblastomas (Furchert *et al*., 2007). Histone deacetylases (HDAC) and histone acetyl transferases modify histone proteins and contribute to an epigenetic code recognized by proteins involved in regulation of gene expression (Marks *et al*., 2003, 2004; Hooven *et al*., 2005). Indeed, former studies demonstrated the cytotoxicity of HDAC inhibitors to several neuroblastoma cells, resulting in growth inhibition of these tumor cells (Cinatl *et al*., 1996; Michaelis *et al,* 2004, 2007; Furchert *et al*., 2007). In neoplastic cells, where overexpression of different HDACs was frequently detected (for summary see, Bolden *et al,* 2006), the abundance of deacetylated histones is usually associated with DNA hypermethylation and gene silencing (Santini *et al*., 2007). Treatment with HDAC inhibitors induced the reactivation of growth regulatory genes and consequently apoptosis in these cells. One of the HDAC inhibitors, valproic acid (VPA), inhibits growth and induces differentiation of human neuroblastoma UKF-NB-2 and UKF-NB-3 cells *in vitro* at concentrations ranging from 0.5 to 2 mM that have been achieved in human with no significant adverse effects (Cinatl *et al*., 1996). However, information on effects of VPA and other HDAC inhibitors on additional neuroblastoma cells are scarce. Therefore, here we extended this study by investigating the effect of VPA and another HDAC inhibitor, trichostatin A (TSA), on other neuroblastoma cell lines. Because heterogeneity of neuroblastoma cells could affect their treatment, two types of neuroblastoma cell lines were tested for their response to VPA and TSA treatment. Besides the effect of VPA and TSA on UKF-NB-3 cells (the invasive N-type), that on the UKF-NB-4 and SK-N-AS cell lines (the non-invasive and less-aggressive S-type) was investigated in this work.

In addition, VPA and TSA are known to be metabolized by cytochrome P450 (CYP) biotransformation enzymes and can increase and/or decrease their activities and/or expression, thereby affecting mechanisms that control drug disposition (Fisher *et al*., 1991; Rogiers *et al*., 1992, 1995; Isojärvi *et al*., 2001; Wen *et al*., 2001; Bort *et al*., 2004; Cerveny *et al*., 2007; Nelson-DeGrave *et al*., 2004; Hooven *et al*., 2005; Snykers *et al*., 2007; Kiang *et al*., 2006). Because several CYP enzymes metabolizing a variety of drugs (CYP1A1, 1B1 and 3A4) were found to be expressed in neuroblastoma cells (Poljaková *et al*., 2009), here we also investigated whether their expression is influenced by VPA and TSA in these cells.

## Material and methods

### Chemicals

Valproate and trichostatin A were obtained from Sigma (St. Louis, MO, USA). All other chemicals used in the experiments were of analytical purity or better.

### Cell cultures

The UKF-NB-3 and UKF-NB-4 neuroblastoma cell lines, established from bone marrow metastases of high-risk neuroblastoma, were a gift of prof. J. Cinatl, Jr. (J. W. Goethe University, Frankfurt, Germany). Cell line UKF-NB-4 was established from infiltrated bone marrow of chemoresistant high-risk neuroblastoma recurrence and have high expression of P-glycoprotein. SK-N-AS, derived from bone marrow metastases of neuroblastoma, was of the commercial source (ECACC, Salisbury, UK). Cells were grown at 37°C and 5% CO_2_ in Iscove's modified Dulbecco's medium (IMDM) (KlinLab Ltd, Prague, Czech Republic), supplemented with 10% fetal bovine serum, 2 mM L-glutamine, 100 units/ml of penicilline and 100 µg/ml streptomycine (PAA Laboratories, Pasching, Austria).

### MTT assay

The cytotoxicity of valproate and trichostatin A was determined by MTT test. For a dose-response curve, culture medium stock solutions of valproate (200 mM) and DMSO solutions of trichostatin A (1 mM) were dissolved in culture medium to final concentrations of 0 – 50 mM and 0 – 1 µM for valproate and trichostatin A, respectively. Cells in exponential growth were seeded at 1×10^4^ per well in a 96-well microplate. After incubation (72 hours) at 37°C in 5% CO_2_ saturated atmosphere the MTT solution (2 mg/ml PBS) was added, the microplates were incubated for 4 hours and cells lysed in 50% N,N-dimethylformamide containing 20% of SDS, pH 4.5. The absorbance at 570 nm was measured for each well by multiwell ELISA reader Versamax (Molecular devices, CA, USA). The mean absorbance of medium controls was subtracted as a background. The viability of control cells was taken as 100% and the values of treated cells were calculated as a percentage of control. The IC_50_ values were calculated from at least 3 independent experiments using linear regression of the dose-log response curves by SOFTmaxPro.

### Estimation of contents of cytochromes P450 1A1, 1B1 and 3A4 in neuroblastoma cells by Western blot

To determine the expression of CYP1A1, 1B1 and 3A4 proteins, cells were homogenized in RIPA buffer. Protein concentrations were assessed using the DC protein assay (Bio-Rad, Hercules, CA, USA) with serum albumin as a standard. 10–45 µg of extracted proteins were subjected to SDS-PAGE electrophoresis on a 10% gel. After migration, proteins were transferred to a nitrocellulose membrane and incubated with 5% non-fat milk to block non-specific binding. The membranes were then exposed to specific anti-CYP1A1 (1:1000, Millipore, MA, USA) anti-CYP1B1 (1:500, AbCam, MA, USA) and anti-CYP3A4 (1:5000, AbD Serotec, Oxford, UK) rabbit polyclonal antibodies overnight at 4°C. Membranes were washed and exposed to peroxidase-conjugated anti-IgG secondary antibody (1:3000, Bio-Rad, Hercules, CA, USA), and the antigen-antibody complex was visualized by enhanced chemiluminiscence's detection system according to the manufacturer's instructions (Immun-Star HRP Substrate, Bio-Rad, Hercules, CA, USA). Films (MEDIX XBU, Foma, Hradec Králové, Czech Republic) were scanned with a computerized image-analyzing system (ElfoMan 2.0, Ing. Semecký, Prague, Czech Republic).

## Results

### Cytotoxicity of valproate and trichostatin A to human neuroblastoma cells

**Figure 1:**
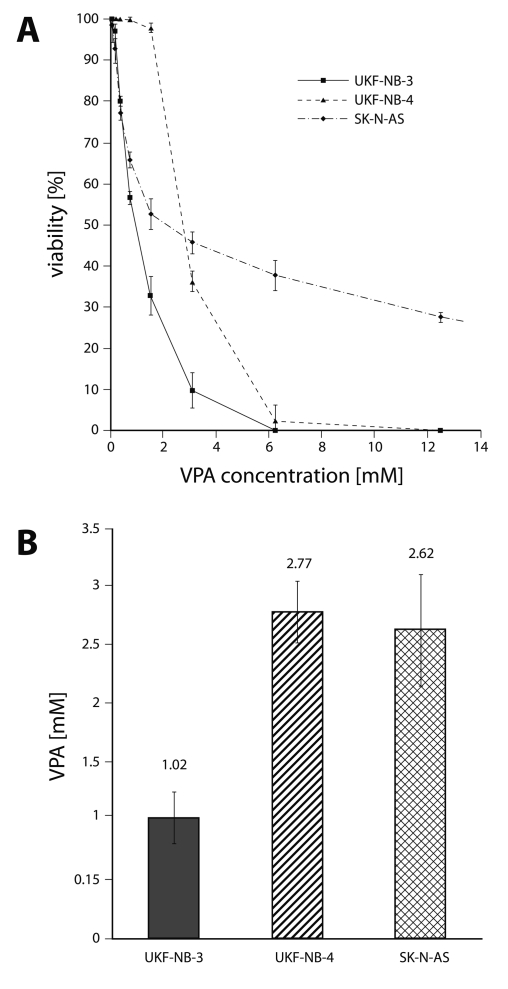
Cytotoxicity (viable cells as percentage of control) of valproate to UKF-NB-3, UKF-NB-4 and SK-N-AS after 72 h exposure to the compound, determined by the MTT assay (**A**) and the values of IC_50_ (**B**). Values are means and standard deviations of 8 determinations.

**Figure 2:**
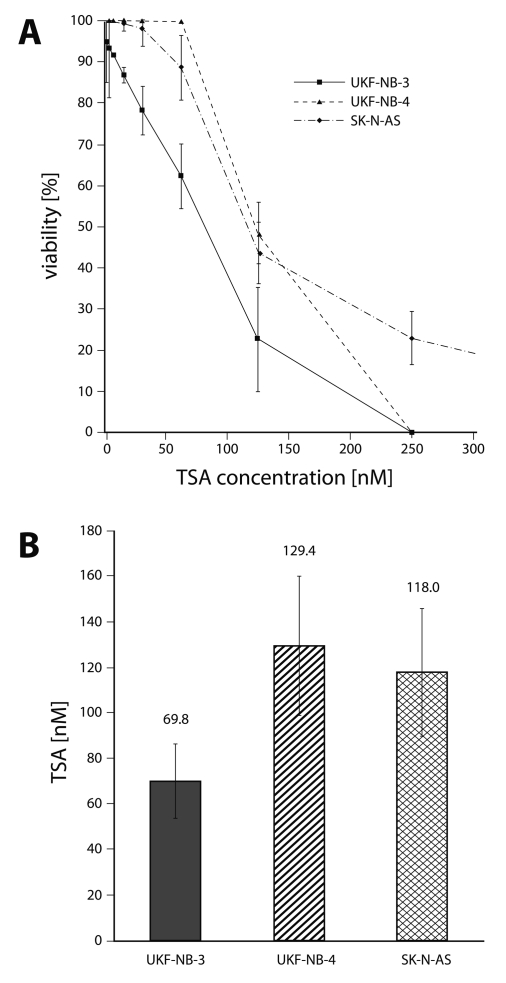
Cytotoxicity (viable cells as percentage of control) of trichostatin A to UKF-NB-3, UKF-NB-4 and SK-N-AS after 72 h exposure to the compound, determined by the MTT assay. (**A**) and the values of IC_50_ (**B**). Values are means and standard deviations of 8 determinations.

To evaluate the cytotoxicity of VPA and TSA to human neuroblastoma cells (UKF-NB-3, UKF-NB-4 and SK-N-AS), these cells were treated with increasing concentrations of both drugs (0–50 mM for VPA and 0–1 µM for TSA). We first determined the effect of VPA and TSA on growth of human neuroblastoma cell lines cultured for 72 hours in the presence of both drugs, using MTT assay. As shown in Figures 1 and 2, all three neuroblastoma cell lines were sensitive to VPA and TSA. Both drugs inhibited the growth of neuroblastoma cell lines in a dose-dependent manner. The IC_50_ values for VPA and TSA calculated from the dose-log response curves are shown in Figures 1B and 2B.

Among the neuroblastoma cell lines tested in this study, the UKF-NB-3 cell line was the most sensitive to both drugs, with IC_50_ values of 1.02 mM and 69.8 nM for VPA and TSA, respectively. The IC_50_ values indicating the toxicity of VPA and TSA to UKF-NB-4 cells were similar to those found for the SK-N-AS cell line, being up to a 2.7-fold lower than for the UKF-NB-3 cell line (Figures 1 and 2). Nevertheless, the curves showing the viability of SK-N-AS cells under treatment with increasing concentrations of VPA and TSA significantly differed from those of UKF-NB-3 and UKF-NB-4 cell lines. At higher VPA and TSA concentrations, the sensitivity of SK-N-AS cells was much lower than that of other two neuroblastoma cell lines analyzed in this work.

### Effect of VPA and TSA on expression of cytochrome P450 1A1, 1B1 and 3A4 proteins

Using Western blot analysis with antibodies raised against CYP1A1, 1B1 and 3A4, the effects of VPA and TSA on protein expression levels of these enzymes were analyzed in the tested neuroblastoma cell lines.

**Figure 3:**
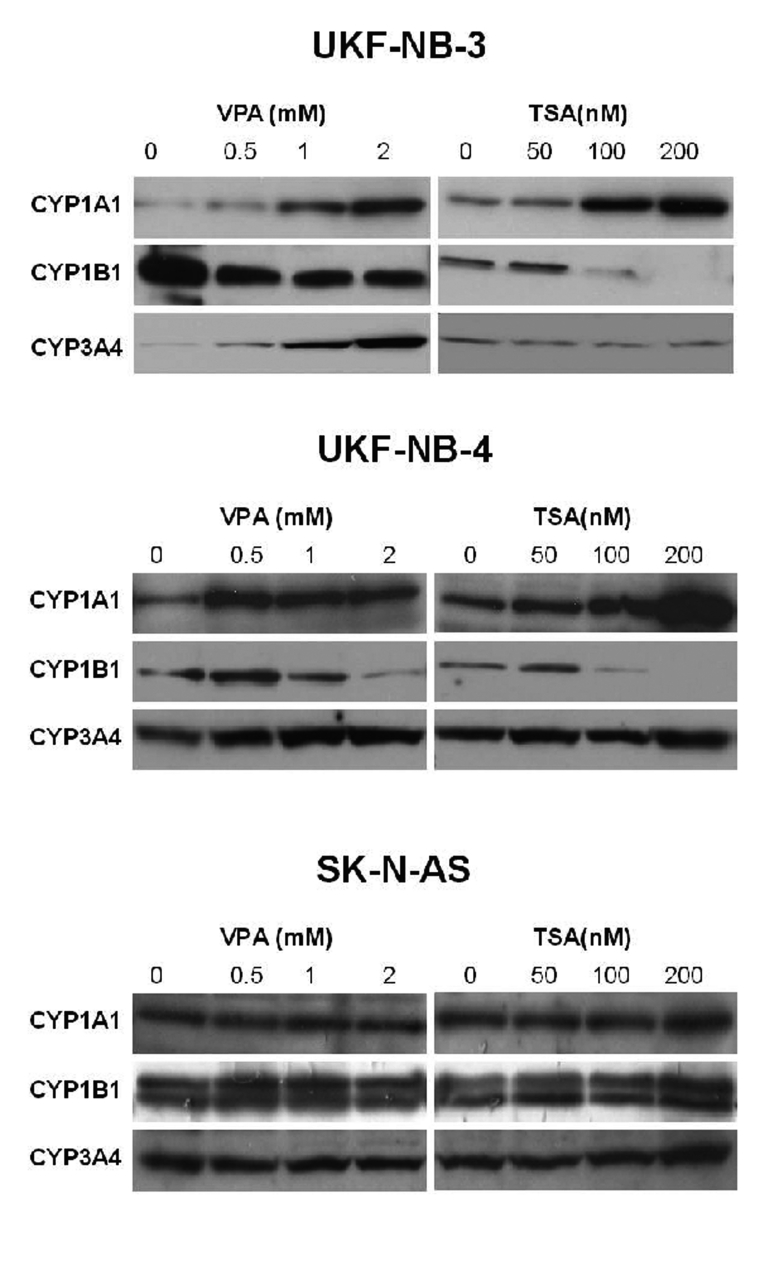
Expression of CYP1A1, 1B1 and 3A4 in human neuroblastoma cell lines UKF-NB-3, UKF-NB-4 and SK-N-AS by Western blot.

Expression of CYP1A1 protein in neuroblastoma UKF-NB-3 and UKF-NB-4 cells was elevated by increasing concentrations of VPA and/or TSA in a dose-dependent manner (Figure 3). A 1.7-, 4.0- and 8.1-fold increase in CYP1A1 expression was caused by treating the UKF-NB-3 cells for 48 hour with 0.5, 1.0 and 2.0 mM VPA, respectively, while lower, only up to a 1.7-fold increase in levels of this CYP was produced by VPA in UKF-NB-4 cells. In the SK-N-AS cells, even no effect of VPA on the CYP1A1 expression was detectable.

Similar effects on CYP1A1 expression in neuroblastoma UKF-NB-3 and UKF-NB-4 cell lines were detected when cells were treated for 48 hours with TSA. Up to a 4.4-fold increase in expression levels of CYP1A1 was produced by 50–200 nM TSA in these cells (Figure 3). No effects of TSA on the expression of CYP1A1 protein in SK-N-AS were found.

Expression of CYP1B1 protein was decreased in UKF-NB-3 and UKF-NB-4 cells after their 48-hour treatment with increasing concentrations of VPA and/or TSA, being decreased in a dose-dependent manner. Similar to CYP1A1, no effect of both HDAC inhibitors on expression of CYP1B1 was produced in SK-N-AS cells (Figure 3). Interestingly, two protein bands detectable by antibody against CYP1B1 were found in SK-N-AS cells.

In the case of the effects of VPA and TSA on expression of CYP3A4 protein in neuroblastoma cells, both these drugs essentially did not influence its expression in S-type UKF-NB-4 and SK-N-AS cell lines. The N-type of neuroblastoma cell line, UKF-NB-3, was only the exception; whereas increased concentrations of VPA increased CYP3A4 expression in this cell line, TSA had no effect (Figure 3).

## Discussion

The results of this work show that human neuroblastoma UKF-NB-3, UKF-NB-4 and SK-N-AS cell lines are sensitive to two tested HDAC inhibitors, VPA and TSA. In the case of VPA, its concentrations that were toxic to neuroblastoma cells are clinically applicable, since concentrations between 0.35–0.7 mM in patients serum are commonly therapeutically used (Duenas-Gonzalez *et al*., 2007). The IC_50_ values for VPA and TSA indicate that a UKF-NB-3 cell line was the most sensitive to both HDAC inhibitors, while their toxicity to the UKF-NB-4 and SK-N-AS cell lines was up to a 2.7-fold lower. Thus, the sensitivity to the two drugs seems to be related to the phenotype, with the S-type cells (UKF-NB-4 and SK-N-AS) being less sensitive than the N-type (UKF-NB-3), probably because of their partly lower capability of undergoing apoptosis (Servidei *et al*., 2004). However, the results shown here indicate that it seems to be questionable to evaluate the toxic effects of chemicals to cells in culture using only the IC_50_ values. The question arises, whether the IC_50_ value is a real appropriate sensitivity marker. Namely, of the S-type neuroblastoma cells utilized in this study, the SK-N-AS cell line seems to be even less sensitive to VPA and TSA than the second S-type cell line, UKF-NB-4, even though the IC_50_ values for VPA and TSA were similar for both these cell lines. At higher VPA and TSA concentrations, the sensitivity of SK-N-AS cells was much lower than that of UKF-NB-4. This less sensitive SK-N-AS line seems to be, at least in part, capable of overcoming treatment with VPA and TSA at concentrations that cause almost complete eradication of UKF-NB-4 cells. These results suggest that caution should be exerted to sort neuroblastoma cells into their types. Even in one type of neuroblastoma cells (S-type in this case), biological heterogeneity should be taken into account. This suggestion is also supported by further features found in this cell line. Namely, the SK-N-AS cell line behaves differently from the other S-type cell line, UKF-NB-4, from the point of view of the effects of VPA and TSA on CYP expression; no effects of both drugs was found on levels of individual CYP enzymes. Moreover, in this cell line, the two CYP1B1 protein bands were detectable by antibody against this CYP. Now, we can only speculate on the origin of the second protein band in SK-N-AS cells. The questions, whether it might follow from a degradation of the CYP1B1 protein in this cell line or it is the artifact caused by the method used (Western blot), remain to be answered.

The expression of all CYP enzymes analyzed in this work was modulated by VPA and TSA only in the N-type UKF-NB-3 cell line. Whereas the CYP1A1 enzyme was induced by both drugs, expression of CYP1B1 was depressed by both drugs. The CYP3A4 enzyme was increased by VPA, but TSA had no influence on the expression of this enzyme. The expression of CYP1A1 and 1B1 was also similarly affected by VPA and TSA in the UKF-NB-4 cell line, but no effect on expression levels of CYP3A4 was produced in this line. Similarity in response of UKF-NB-3 and UKF-NB-4 cells to the effects of VPA and TSA on CYP1A1 and 1B1 expression might probably be caused by their similar effects on state (degree) of acetylation of histones and, therefore, transcription activity. But differences between these two cell lines and particularly SK-N-AS cells in response to CYP enzyme expression and its affecting by VPA and TSA are still valuable. The question whether such differences are caused by the fact that cells vary in the broad spectrum of metabolic and signalling pathways that might also be affected by VPA and TSA in a different way, independently of a cell type (N- or S-type), remains to be answered. Further studies with these and other neuroblastoma cell lines and various HDAC inhibitors and broader spectrum of CYP enzymes have to be performed in order to shed more light on this field.

Since CYP enzymes are involved in biosynthesis and metabolism of many endogenous physiologically active substances and in biotransformation of xenobiotics with pharmacological and/or toxic effects (Myasodeova, 2008), a change in their expression might affect the cells significantly. In the case of oncology, the participation of CYPs in drug metabolism seems to be their most important role. A variety of CYP enzymes is involved in metabolism of a broad spectrum of drugs that can, moreover, either increase or decrease their expression levels. The finding that VPA and TSA are capable of inducing and depressing CYP enzyme expression in neuroblastoma cells (CYP1A1, 1B1 and 3A4 tested in our work) might have great importance. This feature might be utilized mainly in the combination therapy with other drugs whose pharmacological effects are dependent on their CYP-mediated metabolism. Such a study with one of these drugs, ellipticine, is under way in our laboratory.
